# Effect of Sulfonate-, Phosphonate-, and Lipoate-Functional Modifiers on the Mechanical and Thermal Properties of Polymethyl Methacrylate Denture Base Resin

**DOI:** 10.3390/polym18202472

**Published:** 2026-10-10

**Authors:** Selda Gökçe Erdal, Merve Acar, Ayşegül Köroğlu, Baki Hazer

**Affiliations:** 1 Department of Prosthodontics, Faculty of Dentistry, Zonguldak Bülent Ecevit University, 67100 Zonguldak, Türkiye; 2 Department of Aircraft Airframe Engine Maintenance, Kapadokya University, 50420 Ürgüp, Türkiye; 3 Department of Chemistry, Zonguldak Bülent Ecevit University, 67100 Zonguldak, Türkiye

**Keywords:** poly(methyl methacrylate), denture bases, lipoate, bisphosphonate, sodium 4-styrenesulfonate, flexural strength, thermal stability

## Abstract

**Objective**: This study aimed to evaluate the mechanical and thermal properties of heat-polymerized polymethyl methacrylate (PMMA) denture base resin modified with different concentrations of sodium 4-styrenesulfonate (SO), 5,5-bis(diisopropoxyphosphoryl)pentyl methacrylate (PO), and 5,5-bis(diisopropoxyphosphoryl)pentyl lipoate (LIP). **Materials and Methods**: Heat-polymerized PMMA specimens were divided into a control group and experimental groups modified with 2%, 3%, or 5% SO, PO, or LIP (n = 8 per group). Maximum deflection, flexural strength, and Young’s modulus were determined using a three-point bending test. Surface hardness was evaluated using the Vickers hardness test, thermal behavior was assessed by thermogravimetric analysis (TGA), and fracture surfaces were examined by scanning electron microscopy (SEM). Data were analyzed using two-way ANOVA (modifier type × concentration) with Bonferroni-adjusted pairwise comparisons, and comparisons including the control group were performed using Welch’s or one-way ANOVA followed by Tamhane’s T2 or Bonferroni post hoc tests, depending on the homogeneity of variances (α = 0.05). **Results**: Modifier type significantly affected maximum deflection, flexural strength, Young’s modulus, and Vickers hardness of specimens. The LIP-containing groups showed the highest mean deflection values but significantly lower flexural strength, Young’s modulus, and hardness than the control group. In contrast, the SO-containing groups generally maintained mechanical properties closer to those of unmodified PMMA. TGA showed that all modifiers altered the thermal degradation profile of PMMA, with PO increasing the main degradation temperature by approximately 40 °C. **Conclusions**: The investigated modifiers produced distinct, modifier-dependent changes in the mechanical and thermal behavior of heat-polymerized PMMA. Although LIP increased deformation capacity, this occurred at the expense of flexural strength and stiffness. SO and PO modifications showed comparatively smaller changes in mechanical properties at selected concentrations. Further studies including direct fracture-toughness, fatigue, and clinically relevant aging assessments are required to determine whether these modifications provide functional advantages for denture base applications.

## 1. Introduction

Conventional removable dentures remain the treatment of choice for many edentulous patients due to medical and financial constraints, despite the increasing use of dental implants for oral rehabilitation [1]. In this context, the synthetic polymer poly(methyl methacrylate) (PMMA), which is classified by the IUPAC system as poly [1-(methoxycarbonyl)-1-methylethylene] and synthesized via free radical chain polymerization of methyl methacrylate monomers, continues to be widely used [2]. Despite the development of various alternative polymers for denture base fabrication, PMMA retains its status as the most widely used material, as no alternative simultaneously meets all the necessary criteria, including mechanical properties, processability, favorable esthetics, and cost-effectiveness [3]. However, its limited mechanical strength predisposes denture bases to crack formation and fracture, particularly under long-term clinical use [4]. Denture fractures are more frequently observed in elderly patients, largely due to age-related decline in neuromuscular control and the associated risk of accidental dropping [5].

In the literature various strategies have been investigated to reduce the fracture risk of PMMA denture bases, including the metal framework usage, addition of filler materials, dental fibers and nanoparticles, the use of novel fabrication techniques such as injection molding and the modification of polymer chemistry through copolymerization or cross-linking [1,5].

Through chemical modification approaches aimed at enhancing the mechanical and physical properties of PMMA—specifically, by incorporating various monomers or functional compounds into the polymer structure—copolymers that influence the material’s clinical performance can be obtained [3,6]. Accordingly, compounds carrying functional groups that differ in polarity, acidity, and polymerization behavior can help clarify how the chemistry of a modifier translates into changes in the properties of PMMA. In this context, sulfur- and phosphorus-containing compounds are of particular interest, as their functional groups can influence both the intermolecular interactions and the thermal behavior of polymers. Sulfonate-containing polymers have been widely studied owing to the high polarity and distinctive chemical properties of the sulfonate group [7,8]. Phosphonate-containing compounds have been investigated as functional components of polymeric systems [9,10] and have been shown to modify the thermal degradation behavior of PMMA [11,12]. Lipoic acid-based compounds, in turn, have attracted growing interest in polymer science because of the distinctive reactivity of their disulfide-containing ring structure [13,14,15]. Comparing modifiers that share the same functional group but differ in the unit through which they are intended to be incorporated into the polymer, together with a modifier bearing a different functional group, may help distinguish the contribution of the functional group from that of the polymerizable unit. However, information on sulfur- and phosphorus-containing modifiers on the properties of denture base PMMA remains limited.

The aim of this study was to comparatively evaluate the effects of incorporating a sulfonate monomer (SO) and two bisphosphonate ester–functional modifiers sharing the same side group, a methacrylate (PO) and a lipoate (LIP), at different concentrations on the surface hardness, flexural strength, Young’s modulus, and thermal stability of heat-polymerized PMMA denture base resin. The null hypothesis was that the incorporation of SO, PO, or LIP at varying concentrations would not significantly affect the surface hardness, flexural strength, Young’s modulus, or thermal stability of PMMA compared with the control group.

## 2. Materials and Methods

### 2.1. Synthesis of Modifiers

**Figure 1:**
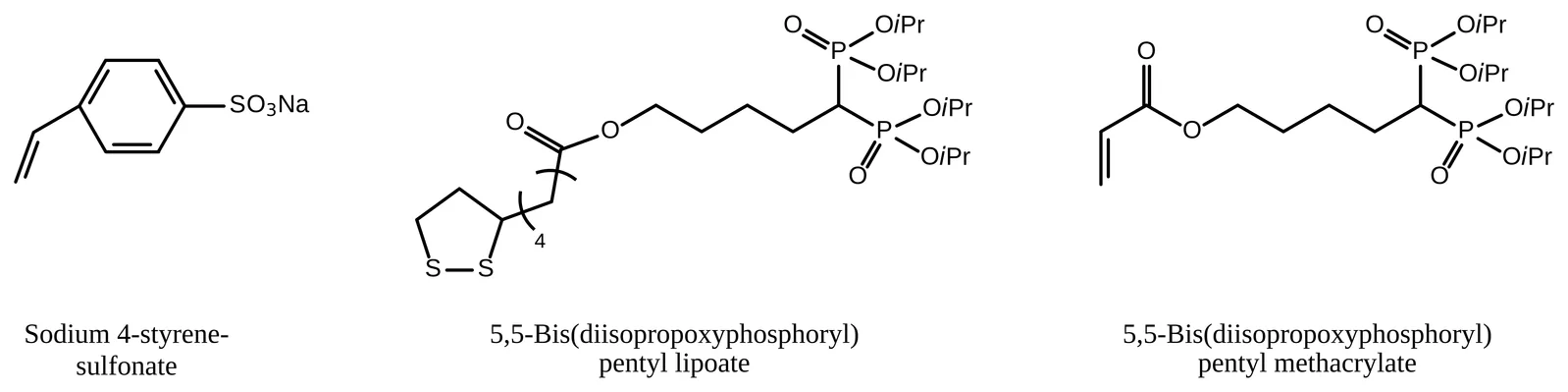
Chemical structures of sodium 4-styrenesulfonate (SO), 5,5-bis(diisopropoxyphosphoryl)pentyl lipoate (LIP), and 5,5-bis(diisopropoxyphosphoryl)pentyl methacrylate (PO) incorporated into the PMMA matrix.

The following reagents were purchased and used without further purification: sodium 4-styrenesulfonate (SO), methacryloyl chloride, and α-lipoic acid (LA) from Sigma-Aldrich (St. Louis, MO, USA), and tetraisopropyl methylenediphosphonate (Tiph) from Sigma-Aldrich (Taufkirchen, Germany). The modifiers PO and LIP were synthesized in our laboratory from these starting materials, as described below (Figure 1).

#### 2.1.1. Synthesis of 5,5Bis(diisopropoxyphosphoryl)pentyl Methacrylate (PO)

5,5Bis(diisopropoxyphosphoryl) pentyl methacrylate (PO) was synthesized according to the procedure reported in cited article [16]. Briefly, the synthesis was carried out in three steps. In the first step bis(diisopropoxyphosphoryl)pentyl acetate was obtained (Tiph-A). For this NaH (1.55 g, 35 mmol of 60 wt.%) was suspended in DMF (80 g) and stirred at 0 °C for 1 h. Tiph (9.6 g, 27.9 mmol) was added dropwise at 0 °C. After 1 h, For this purpose, 4-Br-butyl acetate (10 g) was added dropwise at 0 °C. It was stirred for 1 h. The reaction was continued at 60 °C for 24 h. Aqueous NaCl was added to quench the reaction and subsequent extraction of ethyl acetate. The organic phases were combined and washed five times with aqueous NaCl solution. The organic phase was dried on anhydrous Na_2_SO_4_ overnight. Solvents were evaporated under vacuum. The pure product (Tiph-A) was obtained by passing neutral Al_2_O_3_ column as a colorless oil (5.3 g, 41%).

In the second step, Tiph-A was converted to the corresponding hydroxyl derivative, tetraisopropyl (5-hydroxypentane-1,1-diyl)bis(phosphonate) (Tiph-OH). For this purpose, 5,5-bis(diisopropoxyphosphoryl)pentyl acetate (3 g, 6.54 mmol) was dissolved in methanol (30 mL) under an argon atmosphere, followed by the addition of potassium carbonate (1.08 g, 7.86 mmol). The obtained mixture was stirred at room temperature for 24 h. Methanol was evaporated and the product was extracted by ethyl acetate; organic phases were combined and washed with a saturated aqueous solution of NaCl and dried over Na_2_SO_4_. The solvent was removed under reduced pressure. The pure product was obtained as a colorless oil (2.53 g, 93%) and used in the next step without additional purification.

In the final step, methacryl group was attached to the hydroxyl group of Tiph-OH (5,5-bis(diisopropoxyphosphoryl)pentyl methacrylate, MA-Tiph). Tiph-OH (1 g, 2.4 mmol) was dissolved in CH_2_Cl_2_ (10 mL) under argon atmosphere, followed by the addition of Et3N (0.36 g, 3.61 mmol). Then, the solution was cooled down to 0 °C and methacryloyl chloride (0.33 g, 3.18 mmol) was added dropwise. After complete addition, the cooling bath was removed and the solution was stirred at room temperature for 24 h. The product was extracted with CH_2_Cl_2_, organic phases were combined and washed with a saturated solution of NaCl and dried over Na_2_SO_4_. The solvent was removed under reduced pressure. The pure product was obtained by passing neutral Al_2_O_3_ column as a colorless oil (0.62 g, 55%).

#### 2.1.2. Synthesis of (5,5-Bis(diisopropoxyphosphoryl) Pentyl Lipoate (LIP)

The mixture of Tiph-OH (5.01 g, 12.0 mmol), LA (2.33 g, 11.3 mmol), DMAP (0.28 g, 2.3 mmol), and DCC (1.40 g, 6.8 mmol) in CH_2_Cl_2_ (30 mL) was stirred at room temperature for 24 h. The mixture was then poured into 100 mL of saturated aqueous NaCl solution containing 5 mL of 20% HCl. The resulting liquid product was extracted with CHCl_3_, washed with distilled water, and dried over anhydrous Na_2_SO_4_. The solvent was evaporated, and the product was dried under vacuum at room temperature (yield: 5.2 g) and stored in a refrigerator until further use.

The chemical structure of LIP was characterized by ^1^H NMR spectroscopy (600 MHz; Agilent Technologies, Santa Clara, CA, USA), and its elemental composition, particularly the presence of phosphorus and sulfur, was determined by X-ray photoelectron spectroscopy (XPS; K-Alpha, Thermo Fisher Scientific, East Grinstead, West Sussex, UK).

**Figure 2:**
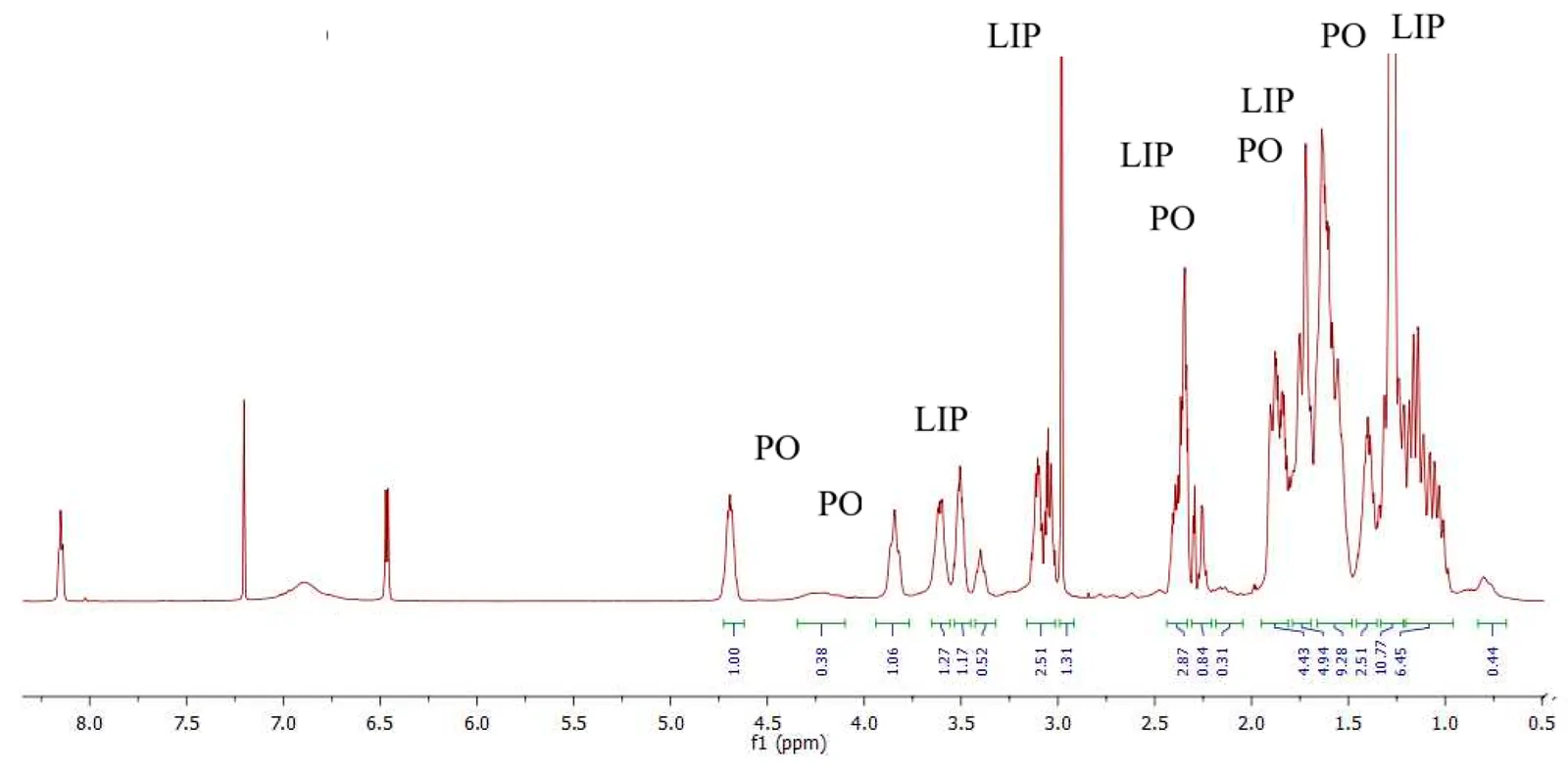
^1^H NMR spectrum of LIP.

XPS analysis confirmed the presence of phosphorus and sulfur in LIP (Table 1). The P 2p and S 2p signals were observed at binding energies of 133.51 and 163.32 eV, respectively, and the phosphorus and sulfur contents were determined as 1.18 and 13.41 wt%, respectively. The ^1^H NMR spectrum of LIP (Figure 2) contained the characteristic signals of both the lipoate and the bisphosphonate ester moieties, confirming the formation of LIP.

### 2.2. Specimen Preparation

In the current study, ten specimen groups were prepared: an unmodified control group and nine experimental groups containing SO, PO, or LIP at concentrations of 2%, 3%, and 5%. Based on a power analysis conducted at a 95% confidence level, a minimum of 7 specimens per group was determined to be sufficient; accordingly, 8 specimens were prepared for each group (n = 80). Heat-polymerized PMMA denture base resin (Imicryl, Konya, Türkiye) was used in this study. The heat-polymerized PMMA resin was used at a powder-to-liquid ratio of 29 g to 10 mL, in accordance with the manufacturer’s recommendations. SO, which was supplied as a powder, was added to the PMMA powder (wt%), whereas PO and LIP, which are liquids, were added to the monomer (vol%). In each case, the modifier replaced the corresponding fraction of the powder or liquid so that the powder-to-liquid ratio recommended by the manufacturer was maintained, and the components were homogenized using a vibrator before mixing.

**Figure 3:**
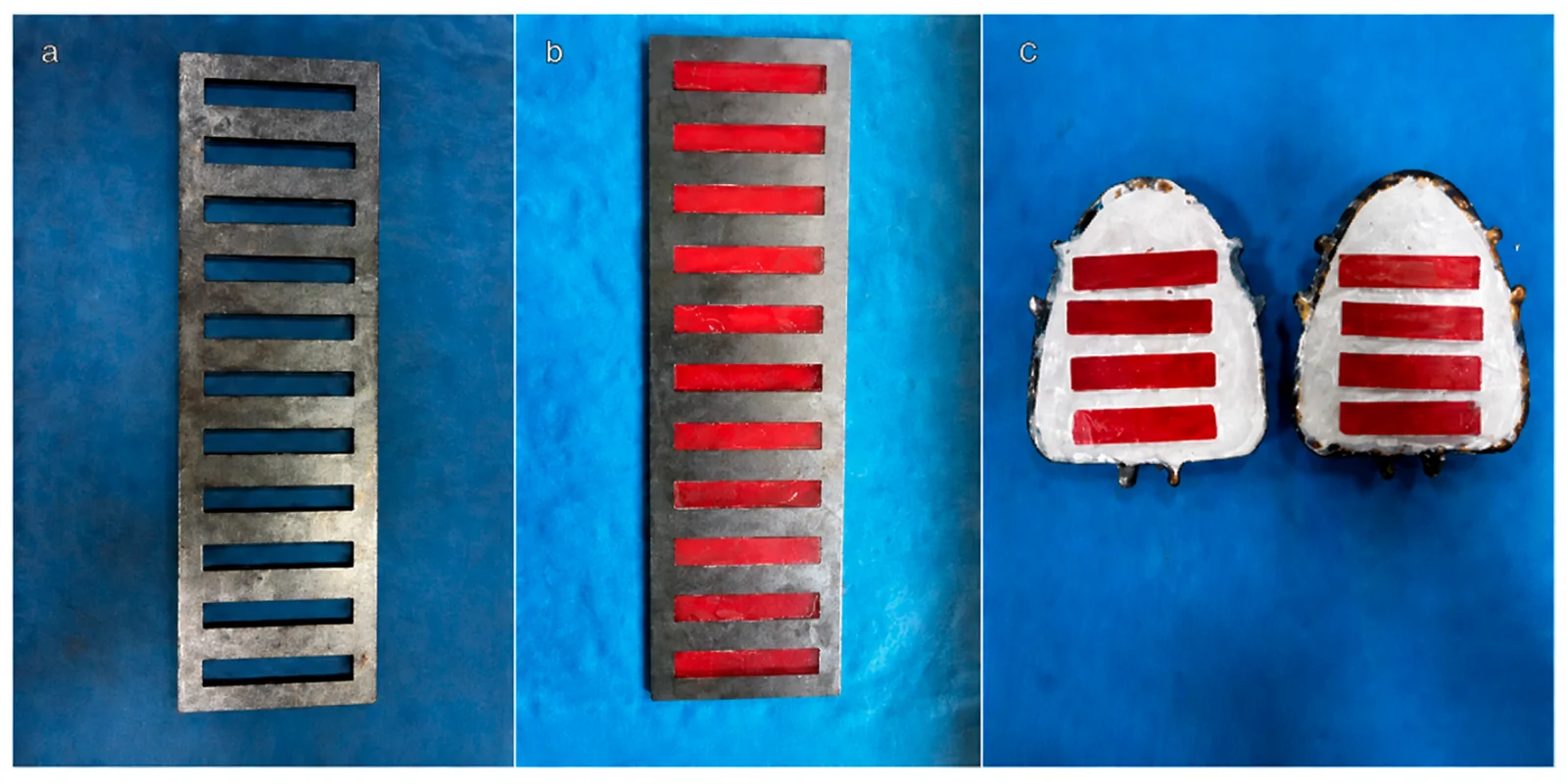
(**a**) Metal mold. (**b**) Wax patterns in metal mold. (**c**) Wax specimens embedded in denture flask.

Rectangular specimens (64 × 10 × 3.3 mm) were fabricated in accordance with ISO 20795-1:2013 [17] using the lost-wax technique. Wax patterns (Cerewax, Pera Dental, İstanbul, Türkiye) were prepared in metal molds to achieve standardized dimensions and were invested in denture flasks (Figure 3). The resin was packed using the conventional compression-molding technique and polymerized in a water bath at 74 ± 1 °C for 2 h. The flasks were bench-cooled before deflasking. To standardize the surface finish, all specimens were sequentially polished with 400-, 600-, and 800-grit silicon carbide abrasive papers using an automated grinder–polisher (Grin PO 2 V, Metkon, Bursa, Türkiye). Before testing, the specimens were stored in distilled water at 37 °C for 50 ± 2 h.

### 2.3. Three-Point Bending Test

**Figure 4:**
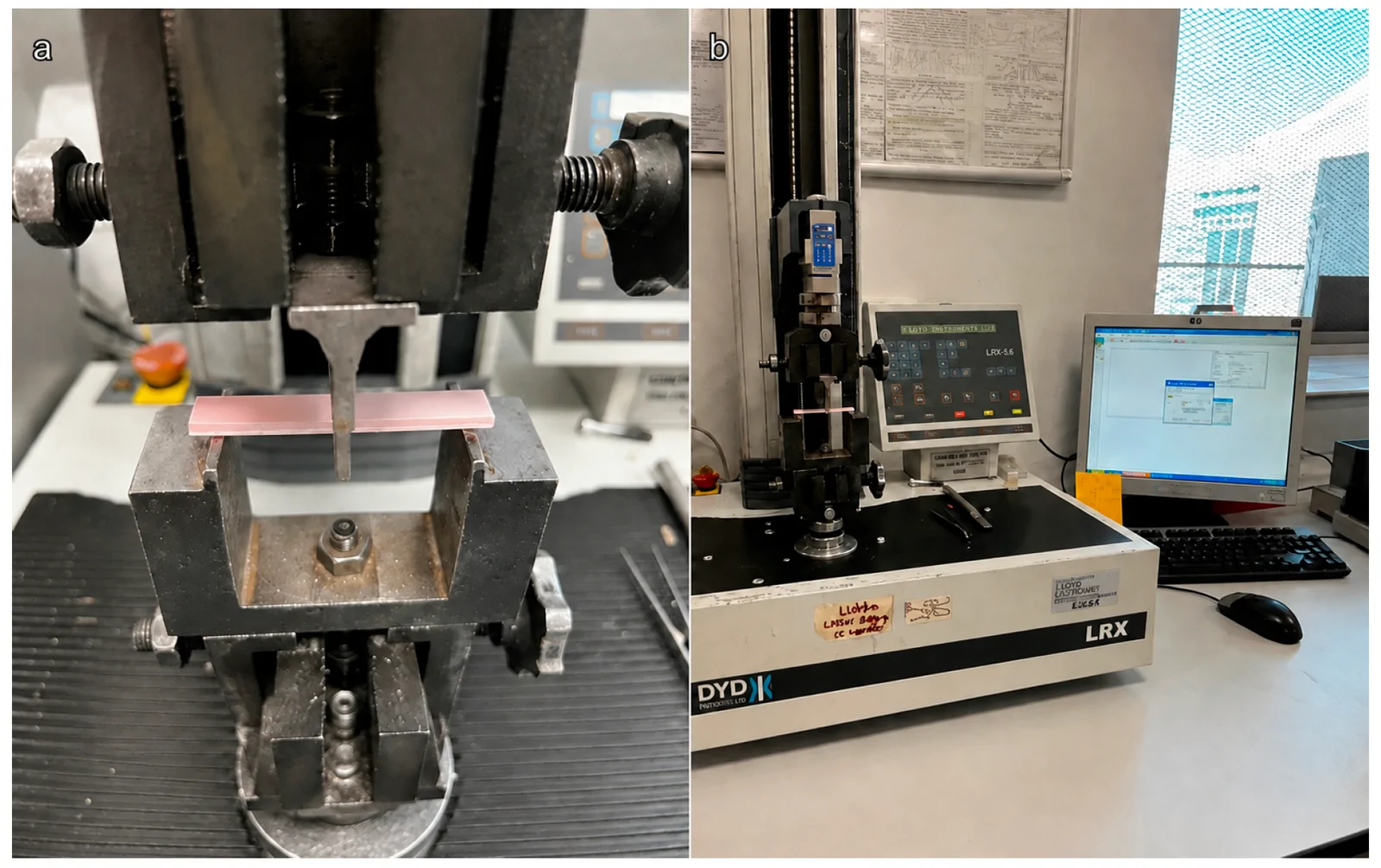
(**a**,**b**) Subjecting the specimens to a three-point bending test.

The flexural strength, Young’s modulus, and deflection at fracture of the specimens were determined by a three-point bending test performed according to the test configuration of ISO 20795-1:2013 using a universal testing machine (Lloyd LRX, Lloyd Instruments, Fareham, UK). The specimens were positioned on two parallel supports with a span length of 50 mm, and a load was applied at the midpoint of each specimen at a crosshead speed of 5 mm/min until fracture (Figure 4).

The flexural strength (σ, MPa) was calculated as σ = 3FL/(2bh^2^), where F is the maximum load at fracture (N), L is the support span (50 mm), b is the specimen width (10 mm), and h is the specimen thickness (3.3 mm).

The Young’s modulus (E, MPa) was calculated as E = FL^3^/(4bh^3^d), where F is a load (N) within the linear elastic region of the load–deflection curve and d is the corresponding deflection (mm).

### 2.4. Surface Hardness

Surface hardness was measured on the fractured fragments after the three-point bending test, in the central region of each fragment, using a Vickers microhardness tester (HMV-G21D, Shimadzu, Kyoto, Japan). A test load of HV0.025 (245.2 mN) was applied for 15 s using a ×40 objective. A total of 11 indentations were made on each specimen, and invalid indentations that could not be measured were excluded, and the mean value was recorded as the hardness of that specimen. Vickers hardness (HV) was calculated as HV = 1.8544 × F/d^2^, where F is the applied load (kgf) and d is the mean diagonal length of the indentation (mm).

### 2.5. Thermal Analysis

Thermal properties of the specimens were evaluated by simultaneous thermogravimetric and differential thermal analysis (TGA–DTA) using a thermal analyzer (TA Instruments, New Castle, DE, USA). Specimens of approximately 10 mg were heated from 30 to 600 °C at a rate of 10 °C/min under a nitrogen atmosphere.

### 2.6. SEM Analysis

The fracture surfaces of representative specimens from each group after the three-point bending test were examined by scanning electron microscopy (Quanta FEG 450, FEI Company, Hillsboro, OR, USA). Prior to analysis, the specimens were mounted on SEM stubs and sputter-coated with gold–palladium (Quorum Q150R ES, Quorum Technologies, Ashford, UK) to enhance conductivity. Images were obtained at 50× and 10,000× magnification.

### 2.7. Statistical Analysis

Statistical analyses were performed using IBM SPSS Statistics version 29.0 (IBM Corp., Armonk, NY, USA). Continuous variables were summarized as mean ± standard deviation (SD), minimum, and maximum values. The normality of the data distribution was assessed using the Shapiro–Wilk test, and the homogeneity of variances was evaluated using Levene’s test. A two-sided *p* value < 0.05 was considered statistically significant.

To evaluate the effects of modifier type (SO, PO, and LIP) and modifier concentration (2%, 3%, and 5%) on maximum deflection, flexural strength, Young’s modulus, and Vickers hardness, the nine modified groups were analyzed using a 3 × 3 two-way factorial ANOVA. The unmodified control group (PMMA_Cnt) was not included in these factorial analyses because it had no modifier type or concentration level. Main effects of modifier type and concentration and the modifier type × concentration interaction were evaluated. When pairwise comparisons were required, Bonferroni-adjusted pairwise comparisons were performed to compare modifier types within each concentration and concentrations within each modifier type.

In addition, the unmodified control group and all nine modified groups were compared using one-way analyses. For maximum deflection, flexural strength, and Vickers hardness, Levene’s test indicated heterogeneity of variances; therefore, overall group differences were evaluated using Welch’s ANOVA, followed by Tamhane’s T2 post hoc test for pairwise comparisons. For Young’s modulus, the homogeneity of variances assumption was satisfied; therefore, one-way ANOVA followed by Bonferroni-adjusted pairwise comparisons was used.

## 3. Results

### 3.1. Thermal Analysis Results

The TGA results are summarized in Table 2. The control group (PMMA_Cnt) exhibited a single-step degradation profile with a Td2 of 350 °C. In contrast, all modified groups showed one or two additional degradation stages at lower temperatures (Td1), indicating a more complex thermal degradation behavior than that of PMMA_Cnt.

In the phosphonate-containing groups (PMMA_PO_2, PMMA_PO_3, and PMMA_PO_5), a single early degradation stage was observed at 229–236 °C, and Td2 ranged from 389 to 393 °C. All phosphonate-containing groups exhibited Td2 values approximately 40 °C higher than that of PMMA_Cnt, whereas Td2 varied only slightly with PO concentration.

The SO-containing groups (PMMA_SO_2, PMMA_SO_3, and PMMA_SO_5) exhibited two early degradation stages, at 235–241 °C and 286–292 °C, and their Td2 values (375–376 °C) were also higher than that of PMMA_Cnt.

The LIP-containing groups (PMMA_LIP_2, PMMA_LIP_3, and PMMA_LIP_5) likewise showed two early degradation stages, at 164–198 °C and 246–249 °C. These groups had the lowest early degradation temperatures among all modified groups, with the lowest value observed in PMMA_LIP_3 (164 °C). Despite this, all LIP-containing groups exhibited higher Td2 values (362–377 °C) than PMMA_Cnt.

Overall, all modified groups exhibited higher Td2 values than the control group; the highest Td2 values were observed in the phosphonate-containing groups, whereas the LIP- and SO-containing groups showed similar values.

### 3.2. Maximum Deflection Results

The effects of modifier type and modifier concentration on maximum deflection were evaluated by two-way ANOVA (Table 3). The main effects of modifier type (F(2, 60) = 31.46, *p* < 0.001) and modifier concentration (F(2, 60) = 4.60, *p* = 0.014) were statistically significant, whereas the interaction between modifier type and modifier concentration was not significant (F(4, 60) = 1.98, *p* = 0.108). This indicates that both modifier type and concentration were associated with differences in maximum deflection, while no significant modifier type × concentration interaction was detected.

Multiple comparisons (Table 4) showed that, at the 3% concentration, PMMA_LIP_3 exhibited significantly greater maximum deflection than PMMA_PO_3 (*p* < 0.05), whereas PMMA_SO_3 did not differ significantly from either group (*p* > 0.05). No significant differences among modifier types were observed at the 2% or 5% concentrations (*p* > 0.05). Within each modifier type, no significant differences were observed among concentrations (*p* > 0.05). Numerically, the highest mean maximum deflection was observed in PMMA_LIP_5 (12.17 ± 4.44 mm), whereas the lowest mean value was observed in PMMA_PO_3 (5.75 ± 1.20 mm).

Descriptive statistics for maximum deflection are presented in Table 5. According to the Shapiro–Wilk test, the data of all groups were normally distributed (*p* > 0.05). Because the homogeneity of variances assumption was violated, as indicated by Levene’s test, between-group differences were evaluated using Welch’s ANOVA, which revealed a significant overall difference among the groups (F(9, 26.301) = 7.411, *p* < 0.001). Pairwise comparisons were subsequently performed using Tamhane’s T2 post hoc test. PMMA_PO_3 exhibited significantly lower maximum deflection than PMMA_Cnt (*p* < 0.05), whereas no significant differences were observed between PMMA_Cnt and the remaining modified groups (*p* > 0.05). In addition, PMMA_LIP_2 showed significantly higher maximum deflection than PMMA_SO_5, and both PMMA_LIP_2 and PMMA_LIP_3 exhibited significantly higher values than PMMA_PO_3 (*p* < 0.05). No other pairwise comparisons were statistically significant (*p* > 0.05).

### 3.3. Flexural Strength Results

The effects of modifier type and modifier concentration on flexural strength were evaluated by two-way ANOVA (Table 6). The main effect of modifier type was statistically significant (F(2, 60) = 32.33, *p* < 0.001), whereas the main effect of modifier concentration was not (F(2, 60) = 2.40, *p* = 0.100). A significant interaction was observed between modifier type and modifier concentration (F(4, 60) = 2.82, *p* = 0.033), indicating that the effect of concentration on flexural strength varied depending on the modifier type.

Multiple comparisons (Table 7) showed that, at the 2% and 5% concentrations, the SO-containing groups exhibited significantly higher flexural strength than the corresponding LIP-containing groups (*p* < 0.05), whereas the PO-containing groups did not differ significantly from either (*p* > 0.05). At the 3% concentration, PMMA_SO_3 exhibited significantly higher flexural strength than both PMMA_PO_3 and PMMA_LIP_3 (*p* < 0.05), while no significant difference was observed between the latter two groups (*p* > 0.05). Within each modifier type, flexural strength did not differ significantly among the tested concentrations (*p* > 0.05). The highest mean value was observed in PMMA_SO_3 (82.80 ± 7.41 MPa), whereas the lowest was recorded in PMMA_LIP_5 (39.59 ± 5.41 MPa).

Descriptive statistics for flexural strength are presented in Table 8. According to the Shapiro–Wilk test, the data of all groups were normally distributed (*p* > 0.05). Because Levene’s test indicated that the homogeneity of variances assumption was violated (F(9, 66) = 2.940, *p* = 0.005), between-group differences were evaluated using Welch’s ANOVA, which revealed a significant overall difference among the groups (F(9, 26.445) = 62.209, *p* < 0.001). Pairwise comparisons were subsequently performed using Tamhane’s T2 post hoc test. PMMA_Cnt exhibited significantly higher flexural strength than PMMA_SO_5, PMMA_PO_3, and all LIP-containing groups (*p* < 0.05), whereas no significant differences were observed between PMMA_Cnt and PMMA_SO_2, PMMA_SO_3, PMMA_PO_2, or PMMA_PO_5 (*p* > 0.05). PMMA_SO_2 exhibited significantly higher values than all LIP-containing groups, PMMA_SO_3 exhibited significantly higher values than PMMA_PO_3 and all LIP-containing groups, and PMMA_SO_5 exhibited significantly higher values than PMMA_LIP_2 and PMMA_LIP_5 (*p* < 0.05). No other pairwise comparisons were statistically significant (*p* > 0.05).

### 3.4. Young’s Modulus Results

The effects of modifier type and modifier concentration on Young’s modulus were evaluated by two-way ANOVA (Table 9). The main effects of both modifier type (F(2, 60) = 53.25, *p* < 0.001) and modifier concentration (F(2, 60) = 4.82, *p* = 0.011) were statistically significant, whereas the interaction between modifier type and modifier concentration was not significant (F(4, 60) = 0.49, *p* = 0.746), indicating that the effect of concentration on Young’s modulus did not vary significantly depending on the modifier type.

Multiple comparisons (Table 10) showed that, at the 2% and 5% concentrations, the SO- and PO-containing groups exhibited significantly higher Young’s modulus values than the corresponding LIP-containing groups (*p* < 0.05), whereas no significant difference was observed between the SO- and PO-containing groups (*p* > 0.05). At the 3% concentration, all modifier types differed significantly from one another, with the highest value observed in PMMA_SO_3, followed by PMMA_PO_3 and PMMA_LIP_3 (*p* < 0.05). Within each modifier type, Young’s modulus did not differ significantly among the tested concentrations (*p* > 0.05). Numerically, the highest mean value was observed in PMMA_SO_3 (2704.51 ± 364.19 MPa), whereas the lowest was recorded in PMMA_LIP_5 (1178.49 ± 147.39 MPa).

Descriptive statistics for Young’s modulus are presented in Table 11. According to the Shapiro–Wilk test, all groups were normally distributed (*p* > 0.05). The homogeneity of variances assumption was satisfied according to Levene’s test (F(9, 66) = 1.898, *p* = 0.068); therefore, the groups were compared using one-way ANOVA. A statistically significant overall difference was observed among the groups (F(9, 66) = 15.783, *p* < 0.001). Bonferroni-adjusted pairwise comparisons showed that PMMA_Cnt exhibited significantly higher Young’s modulus than PMMA_PO_5 and all LIP-containing groups (*p* < 0.05), whereas no significant differences were observed between PMMA_Cnt and the SO-containing groups, PMMA_PO_2, or PMMA_PO_3 (*p* > 0.05). All SO-containing groups exhibited significantly higher values than all LIP-containing groups (*p* < 0.05). Among the SO- and PO-containing groups, significant differences were observed between PMMA_SO_2 and PMMA_PO_5, and between PMMA_SO_3 and both PMMA_PO_3 and PMMA_PO_5 (*p* < 0.05). PMMA_PO_2 and PMMA_PO_3 exhibited significantly higher values than all LIP-containing groups, whereas PMMA_PO_5 differed significantly only from PMMA_LIP_5 (*p* < 0.05). No significant differences were observed among the concentrations within the same modifier type (*p* > 0.05).

### 3.5. Surface Hardness Results

The effects of modifier type and modifier concentration on Vickers hardness were evaluated by two-way ANOVA (Table 12). The main effect of modifier type was statistically significant (F(2, 69) = 93.40, *p* < 0.001), whereas neither the main effect of modifier concentration (F(2, 69) = 1.38, *p* = 0.259) nor the interaction between modifier type and modifier concentration (F(4, 69) = 1.77, *p* = 0.146) was significant, indicating that the effect of concentration on Vickers hardness did not vary according to the modifier type.

Multiple comparisons (Table 13) showed that, at all concentrations, the SO- and PO-containing groups exhibited significantly higher Vickers hardness values than the corresponding LIP-containing groups (*p* < 0.05), whereas no significant differences were observed between the SO- and PO-containing groups at any concentration (*p* > 0.05). Within each modifier type, Vickers hardness did not differ significantly among the tested concentrations (*p* > 0.05). Among the modified groups, the highest mean hardness was observed in PMMA_SO_5 (19.73 ± 3.19 HV), whereas the lowest was recorded in PMMA_LIP_5 (8.68 ± 1.13 HV).

Descriptive statistics for Vickers hardness are presented in Table 14. According to the Shapiro–Wilk test, the data of all groups were normally distributed (*p* > 0.05). Because Levene’s test indicated heterogeneity of variances (F(9, 77) = 2.127, *p* = 0.037), between-group differences were evaluated using Welch’s ANOVA, which revealed a significant overall difference among the groups (F(9, 29.254) = 44.517, *p* < 0.001). Tamhane’s T2 post hoc comparisons showed that PMMA_Cnt exhibited significantly higher hardness than PMMA_PO_2 and all LIP-containing groups (*p* < 0.05), whereas no significant differences were observed between PMMA_Cnt and the SO-containing groups, PMMA_PO_3, or PMMA_PO_5 (*p* > 0.05). All SO- and PO-containing groups exhibited significantly higher hardness than all LIP-containing groups (*p* < 0.05), whereas no significant differences were observed among the SO- and PO-containing groups or among the LIP-containing groups (*p* > 0.05).

### 3.6. SEM Analysis Results

**Figure 5:**
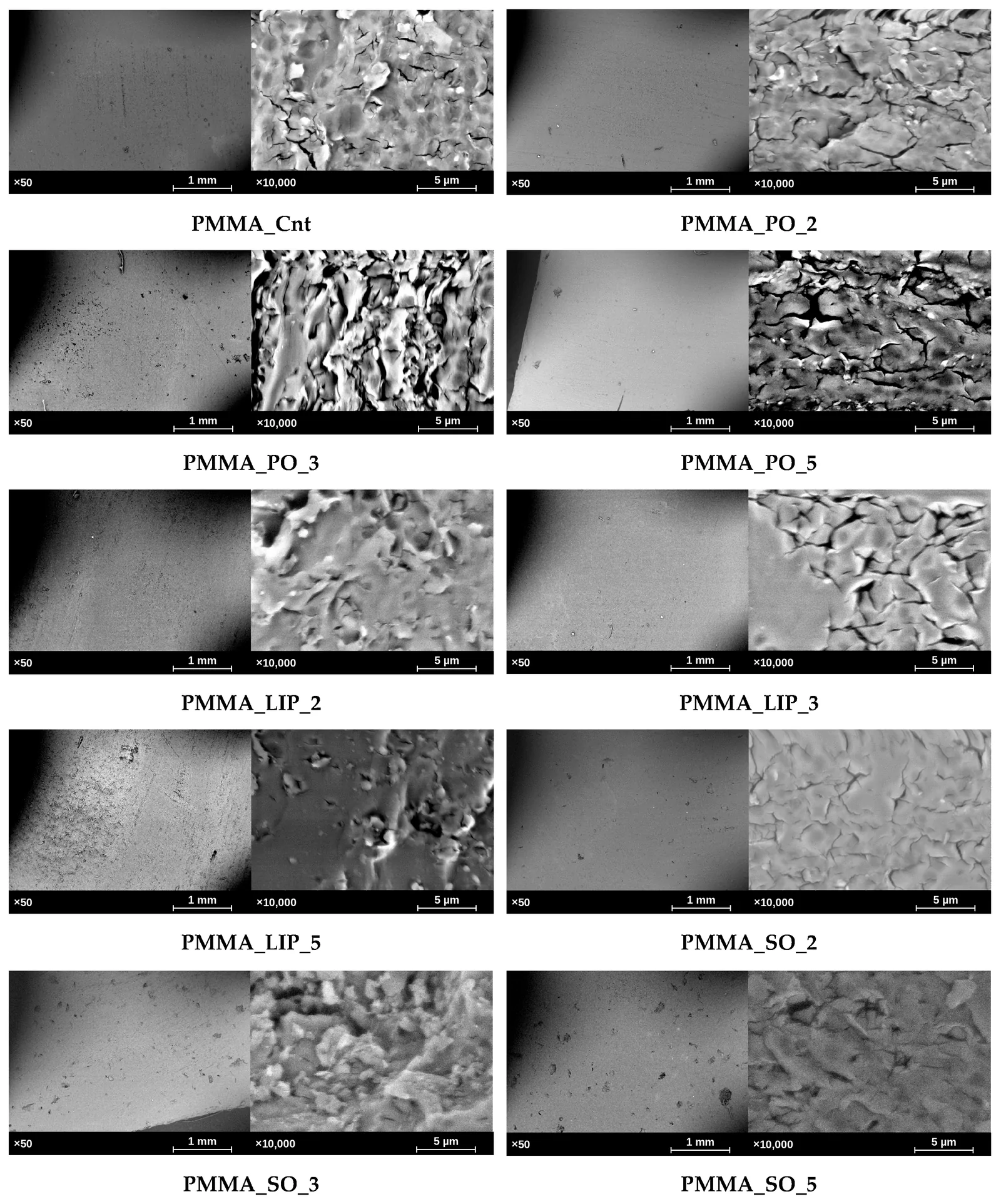
SEM images of the fracture surfaces of the control and modified PMMA groups after the three-point bending test ((**left**), ×50; (**right**), ×10,000).

SEM images of the fracture surfaces revealed qualitative differences in morphology among the groups (Figure 5). At ×50 magnification, all groups exhibited generally smooth surfaces; however, numerous dark spots were observed in PMMA_LIP_5, and fewer scattered spots were present in the SO-containing groups and PMMA_PO_3. At ×10,000 magnification, PMMA_Cnt exhibited a rough fracture surface with multiple cracks and fragmented regions, and the PO-containing groups displayed a similar morphology, with PMMA_PO_3 showing a particularly rough surface with pronounced ridges. In contrast, PMMA_LIP_2 and PMMA_LIP_3 exhibited smoother surfaces with wavy, scale-like features and fewer cracks, whereas PMMA_LIP_5 showed a smooth matrix containing voids and pits. Among the SO-containing groups, PMMA_SO_2 exhibited a relatively smooth surface, whereas PMMA_SO_3 displayed the roughest morphology, with granular, aggregate-like features, and PMMA_SO_5 showed a moderately rough surface.

## 4. Discussion

This study comparatively evaluated the effects of incorporating three chemically distinct modifiers—SO, PO, and LIP—at different concentrations into PMMA on its mechanical and thermal properties.

The null hypothesis, which proposed that these modifications would not significantly affect the surface hardness, flexural strength, Young’s modulus, or thermal stability of PMMA compared with the control group, was partially rejected.

The results demonstrated that the type of modifier and its concentration influenced the material properties in different ways. In particular, LIP markedly reduced surface hardness, flexural strength, and Young’s modulus, while the LIP-containing groups showed the highest mean maximum deflection values, indicating a substantial change in the mechanical behavior of PMMA. In contrast, PO primarily increased the main degradation temperature (Td2) with comparatively limited effects on the mechanical properties, whereas SO exhibited a largely neutral profile and produced results similar to those of the control group across most of the evaluated parameters. These findings indicate that the behavior of the modified PMMA was governed not only by the concentration of the incorporated modifier but also by its chemical structure, suggesting that differences in the chemistry of the modifiers played a decisive role in determining the final material properties.

The mean flexural strength (94.38 MPa) and Young’s modulus (2562.19 MPa) of the PMMA_Cnt group were above the minimum values specified in ISO 20795-1 for heat-polymerizable denture base polymers [17] supporting the reliability of the control group and the test methodology employed in this study.

Although numerous approaches, including fiber reinforcement, nanoparticle incorporation, rubber modification, and copolymerization, have been investigated to improve the mechanical performance of PMMA, no previous study has evaluated lipoate- and bisphosphonate ester–functional modifiers in denture base PMMA or compared them with a sulfonate monomer. Therefore, the present study provides a first comparative evaluation of these modifiers and demonstrates how differences in their molecular structures influence the thermal and mechanical behavior of PMMA.

In the current study, the thermogravimetric analysis results showed that all modified groups exhibited an onset decomposition step at lower temperatures compared with the control group. This finding is in line with previous studies demonstrating that the incorporation of various additives into PMMA can lower the onset decomposition temperature [18,19]. This early degradation stage may also be associated with the volatilization or decomposition of unreacted modifier or other low-molecular-weight species within the matrix.

In particular, the low-temperature onset decomposition step observed in the SO-containing groups supports findings in the literature indicating that sulfonate groups (–SO_3_H) undergo low-temperature dissociation (desulfonation) as the initial thermal degradation event in other polymer systems as well [8].

Notably, the higher Td2 values observed in the PO-containing groups compared with the control group may be attributed to the phosphorus-containing groups, which are known to promote condensed-phase reactions and char formation during thermal degradation, thereby delaying the decomposition of the polymer matrix at elevated temperatures [11,12]. Because thermal stability is governed mainly by these degradation reactions, whereas mechanical properties depend on the network structure and degree of polymerization of the matrix, this increase in Td2 was not accompanied by an improvement in mechanical properties. Although thermal decomposition temperatures in denture base materials occur well above oral physiological temperatures, these parameters are nonetheless regarded as important indicators for assessing material stability during manufacturing, processing, and long-term use.

Lipoate-based polymers have been reported to form through a reversible radical ring-opening polymerization with a ceiling temperature of approximately 139 °C, above which they undergo depolymerization [14]. Consistent with this finding, the LIP-containing specimens in the present study exhibited additional early degradation stages (Td1), suggesting that LIP altered the thermal degradation behavior of PMMA. Previous studies have also demonstrated the beneficial effects of phosphorus-containing functional groups on the thermal behavior of PMMA. Price et al. copolymerized MMA with phosphorus-containing monomers, including a phosphonate methacrylate structurally related to PO, to obtain PMMA derivatives containing approximately 3.5% phosphorus and reported that these copolymers exhibited higher thermal stability than conventional PMMA based on thermogravimetric analysis [11]. Similarly, covalently bound phosphonate groups (2%) have been shown to alter the thermal degradation behavior of PMMA, suggesting that phosphorus-containing functional groups play an important role in improving its thermal stability [12]. These findings are consistent with the present results, in which the PO-containing groups exhibited higher Td2 values than the control group.

The most notable mechanical finding was that LIP modification led to a significant, concentration-independent decrease in Young’s modulus, flexural strength, and surface hardness relative to the control group. The fact that LIP, unlike the other two modifiers, significantly altered the mechanical properties may be related to its limited incorporation into the polymer network, as lipoic acid has been reported to be incompatible with the radical copolymerization of methyl methacrylate [15].

From a clinical perspective, ISO 20795-1 specifies minimum values of 65 MPa for flexural strength and 2000 MPa for flexural modulus for heat-polymerized denture base polymers [17]. Based on the mean values, all SO-containing groups (PMMA_SO_5 only marginally) and PMMA_PO_2 met both requirements; PMMA_PO_3 and PMMA_PO_5 each met only one, and none of the LIP-containing groups met either requirement (flexural strength, 39.59–47.88 MPa; Young’s modulus, 1178.49–1447.88 MPa). Therefore, although the LIP-containing groups exhibited the highest deflection values, this increased deformability was accompanied by a loss of strength and stiffness that places these formulations outside the requirements of the standard. Moreover, increased flexibility does not necessarily translate into improved fracture resistance, which is more directly related to fracture toughness; reinforcement strategies for denture base PMMA, such as the incorporation of fibers and fillers, aim to improve fracture toughness together with strength and stiffness rather than by reducing stiffness [1]. Flexible denture base materials such as polyamides represent a chemically distinct class with their own advantages and limitations [20,21,22,23], and the present findings should not be interpreted as providing an equivalent alternative to these materials.

The present study stated that the LIP-containing groups exhibited the highest maximum deflection values but the lowest surface hardness. Similar studies have reported that the hardness of flexible denture materials is markedly lower than that of both injection-molded and conventionally compression-molded PMMA [20,24], with this reduced hardness attributed to a lower content of cross-linking agents in flexible resins. Although these materials differ chemically from PMMA, a similar inverse relationship between deformability and hardness was observed in the LIP-containing groups.

SO modification did not produce a significant improvement in any of the mechanical parameters examined at the low and moderate concentrations (2% and 3%) compared with the control group, indicating a largely neutral effect on mechanical properties. However, the significant decrease in flexural strength observed at the 5% concentration suggests that this modification may become detrimental at higher concentrations. Accordingly, SO did not appear to confer a clear benefit to PMMA with respect to the properties evaluated in this study, and its use may therefore be justified primarily when a functional benefit beyond mechanical performance—such as antibacterial activity or altered surface properties—is specifically targeted. Interestingly, a previous study incorporating the sodium salt of poly (sodium 4-styrenesulfonate-co-maleic anhydride) (PSSMA) combined with strontium titanate nanoparticles into PMMA reported increases of 48.6% in elastic modulus and 272.8% in microhardness [25]. Although this finding appears, at first glance, to contradict the neutral effect observed for SO in the present study, key methodological differences between the two studies may account for this discrepancy: the sulfonate used in that study was a polymeric compound rather than a monomer, and it was combined with strontium titanate nanoparticles, which may have contributed substantially to the reported improvements.

In a separate study in which methacrylic acid was copolymerized with PMMA to develop a negatively charged denture resin, increasing acid content was associated with a reduction in the load required to fracture the specimens, an effect most pronounced at the highest acid concentration, whereas no significant difference from the control group was observed at the lowest concentration [26]. This finding is consistent with the general trend observed in the present study: a significant reduction in strength was noted in the LIP-containing groups regardless of concentration, whereas most of the SO- and PO-containing groups did not differ significantly from the control group. This suggests that functional modifiers incorporated into PMMA may adversely affect mechanical properties above a certain concentration threshold, depending on their chemical structure.

In the polymer science literature, the effect of functional group incorporation on mechanical properties has been reported to vary depending on polymer structure, with some systems exhibiting an increase in modulus and others a decrease [27]. This provides a supportive framework for explaining why the three modifiers examined in the present study produced effects of differing direction and magnitude on PMMA.

SEM observations of the fracture surfaces qualitatively supported the mechanical test results. The rough, cracked, and fragmented fracture surfaces observed in PMMA_Cnt and the PO-containing groups are consistent with the brittle fracture behavior typical of PMMA. In contrast, the smoother surfaces with wavy, scale-like features observed in PMMA_LIP_2 and PMMA_LIP_3 may indicate a greater degree of deformation before fracture, in agreement with the higher deflection values of these groups. The voids and pits observed in PMMA_LIP_5, together with the dark spots at low magnification, may reflect porosity or phase separation, which could contribute to the low strength and the high variability in deflection observed in this group. Similarly, the granular, aggregate-like features in PMMA_SO_3 and the scattered spots in the SO-containing groups may be related to incomplete dispersion of SO, which was incorporated in powder form. Although SEM analysis alone cannot confirm the underlying mechanisms, these morphological differences are consistent with the mechanical behavior of the respective groups.

This study has certain limitations. As with most PMMA modification studies in the literature [28], the present study was limited to in vitro conditions and does not fully replicate the complex biological and mechanical environment of the oral cavity. Furthermore, only a single commercial heat-cured PMMA system and a limited number of modifier concentrations were evaluated, and parameters that may influence clinical performance—such as fracture toughness, impact strength, water sorption, solubility, color stability, fatigue resistance, and long-term aging—were not investigated. The incorporation of the modifiers into the polymer network was not directly verified. In addition, the three modifiers differed in both their functional groups and polymerizable units, which limits the attribution of the observed effects to a single structural feature. Future studies incorporating different PMMA systems, structurally matched modifiers, structural analyses to verify the proposed mechanisms, fracture toughness testing, and long-term aging protocols would strengthen the clinical relevance of these findings.

## 5. Conclusions

This study demonstrated that the incorporation of SO, PO, and LIP resulted in distinct changes in the thermal and mechanical properties of PMMA. The findings of this study indicate that optimal PMMA modification requires a comprehensive evaluation of mechanical, thermal, and other relevant properties, as an improvement in a single property does not necessarily translate to an overall improvement in material performance; for example, the increased deformability of the LIP-containing groups was accompanied by reduced strength, stiffness, and hardness.

## Figures and Tables

**Table 1:** Elemental composition of LIP determined by XPS.

**Name**	**Peak BE**	**FWHM eV**	**Area (P) CPS.eV**	**Weight %**	**Q**
P2p	133.51	2.49	376.05	1.18	1
S2p	163.32	2.37	5625.80	13.41	1
C1s	284.54	1.92	37838.96	68.44	1
O1s	531.31	2.71	11261.75	11.22	1
N1s	399.36	1.83	4228.08	5.75	1

**Table 2:** TGA results of the PMMA resin groups.

Resin Groups	Td1 (°C)	Td2 (°C)
PMMA_Cnt	-	350
PMMA_PO_2	235	389
PMMA_PO_3	236	391
PMMA_PO_5	229	393
PMMA_SO_2	235, 286	375
PMMA_SO_3	241, 291	375
PMMA_SO_5	238, 292	376
PMMA_LIP_2	198, 249	377
PMMA_LIP_3	164, 246	362
PMMA_LIP_5	190, 246	376

Note: Td1, peak temperature(s) of the early degradation stage(s) in the DTA curve; Td2, peak temperature of the main degradation stage in the DTA curve; -, not observed.

**Table 3:** Two-way factorial analysis of variance results for maximum deflection (mm).

Source	Sum of Squares	df	Mean Square	F	*p*
Modifier type (A)	196.09	2	98.04	31.46	<0.001 *
Modifier concentration (B)	28.68	2	14.34	4.60	0.014 *
A × B	24.73	4	6.18	1.98	0.108
Error	186.96	60	3.12		

Note: The analysis included the nine modified groups; the control group (PMMA_Cnt) was not included. df, degrees of freedom; F, F statistic. * Statistically significant (*p* < 0.05).

**Table 4:** Mean maximum deflection values for the modifier type × concentration interaction (mm; mean ± standard deviation).

Modifier Type	2%	3%	5%
SO	6.75 ± 0.47 ^Aa^	6.52 ± 0.38 ^ABa^	6.50 ± 0.74 ^Aa^
PO	7.25 ± 1.14 ^Aa^	5.75 ± 1.20 ^Ba^	7.20 ± 1.37 ^Aa^
LIP	9.95 ± 1.65 ^Aa^	8.87 ± 1.36 ^Aa^	12.17 ± 4.44 ^Aa^

Note: Within the same column, values not sharing a common uppercase letter differ significantly among modifier types; within the same row, values not sharing a common lowercase letter differ significantly among modifier concentrations (Bonferroni-adjusted pairwise comparisons following two-way ANOVA, *p* < 0.05) SD, standard deviation.

**Table 5:** Descriptive statistics, Shapiro–Wilk normality test, and between-group comparison results for maximum deflection (mm).

Group	Mean ± SD	Minimum	Maximum	Shapiro–Wilk Statistic	Shapiro-Wilk *p*	Welch F	*p*
PMMA_Cnt	7.94 ± 0.78 ^ab^	7.28	9.49	0.812	0.054	7.411	<0.001
PMMA_SO_2	6.75 ± 0.47 ^abc^	6.12	7.42	0.963	0.848		
PMMA_SO_3	6.52 ± 0.38 ^abc^	6.06	7.05	0.938	0.619		
PMMA_SO_5	6.50 ± 0.74 ^bc^	5.02	7.25	0.886	0.213		
PMMA_PO_2	7.25 ± 1.14 ^abc^	6.14	9.56	0.885	0.176		
PMMA_PO_3	5.75 ± 1.20 ^c^	4.02	8.00	0.952	0.713		
PMMA_PO_5	7.20 ± 1.37 ^abc^	5.93	9.74	0.879	0.183		
PMMA_LIP_2	9.95 ± 1.65 ^a^	8.08	12.15	0.873	0.198		
PMMA_LIP_3	8.87 ± 1.36 ^ab^	7.32	10.78	0.926	0.515		
PMMA_LIP_5	12.17 ± 4.44 ^abc^	5.35	17.90	0.968	0.883		
Total	7.79 ± 2.43	4.02	17.90	–	–		

Note: Values are presented as mean ± standard deviation (SD). Different superscript letters indicate statistically significant differences between groups according to Tamhane’s T2 post hoc test (*p* < 0.05); groups sharing at least one common letter are not significantly different. Because Levene’s test indicated heterogeneity of variances, Welch’s ANOVA was used for the overall group comparison, followed by Tamhane’s T2 multiple-comparison test. Normality was assessed using the Shapiro–Wilk test.

**Table 6:** Two-way factorial analysis of variance results for flexural strength (MPa).

Source	Sum of Squares	df	Mean Square	F	*p*
Modifier type (A)	10,385.89	2	5192.95	32.33	<0.001 *
Modifier concentration (B)	770.55	2	385.27	2.40	0.100
A × B	1809.34	4	452.34	2.82	0.033 *
Error	9637.60	60	160.63	—	—

Note: The analysis included the nine modified groups; the control group (PMMA_Cnt) was not included. df, degrees of freedom; F, F statistic. * Statistically significant (*p* < 0.05).

**Table 7:** Mean flexural strength values (MPa) according to modifier type and modifier concentration (mean ± SD).

Modifier Type	2%	3%	5%
SO	78.08 ± 11.94 ^Aa^	82.80 ± 7.41 ^Aa^	65.54 ± 9.17 ^Aa^
PO	70.87 ± 20.67 ^A^ᴮᵃ	55.31 ± 13.51 ^Ba^	65.89 ± 18.45 ^ABa^
LIP	46.54 ± 3.14 ^Ba^	47.88 ± 7.83 ^Ba^	39.59 ± 5.41 ^Ba^

Note: Within the same column, values not sharing a common uppercase letter differ significantly among modifier types; within the same row, values not sharing a common lowercase letter differ significantly among modifier concentrations; simple-effects pairwise comparisons with Bonferroni adjustment following two-way ANOVA, *p* < 0.05. SD, standard deviation.

**Table 8:** Descriptive statistics, Shapiro–Wilk normality test and between-group comparison results for flexural strength (MPa).

Group	Mean ± SD	Minimum	Maximum	Shapiro–Wilk Statistic	Shapiro–Wilk *p*	Welch F	*p*
PMMA_Cnt	94.38 ± 4.94 ^e^	86.83	99.46	0.876	0.211	62.209	<0.001
PMMA_SO_2	78.08 ± 11.94 ^cde^	58.17	93.15	0.956	0.786		
PMMA_SO_3	82.80 ± 7.41 ^de^	71.14	94.37	0.983	0.973		
PMMA_SO_5	65.54 ± 9.17 ^bcd^	48.92	76.47	0.945	0.665		
PMMA_PO_2	70.87 ± 20.67 ^abcde^	42.04	106.41	0.942	0.602		
PMMA_PO_3	55.31 ± 13.51 ^abc^	37.65	74.51	0.908	0.300		
PMMA_PO_5	65.89 ± 18.45 ^abcde^	45.02	99.85	0.936	0.568		
PMMA_LIP_2	46.54 ± 3.14 ^a^	43.23	51.62	0.906	0.371		
PMMA_LIP_3	47.88 ± 7.83 ^ab^	36.72	57.51	0.944	0.673		
PMMA_LIP_5	39.59 ± 5.41 ^a^	31.34	45.86	0.934	0.589		
Total	64.63 ± 19.78	31.34	106.41	–	–		

Note: Values are presented as mean ± standard deviation (SD). Groups that do not share a common superscript letter differ significantly according to Tamhane’s T2 post hoc test (*p* < 0.05); groups sharing at least one common letter are not significantly different. Because Levene’s test indicated heterogeneity of variances, Welch’s ANOVA was used for the overall between-group comparison, followed by Tamhane’s T2 multiple-comparison test. Normality was assessed using the Shapiro–Wilk test. SD, standard deviation.

**Table 9:** Two-way factorial analysis of variance results for Young’s modulus (MPa).

Source	Sum of Squares	df	Mean Square	F	*p*
Modifier type (A)	13,898,784.66	2	6,949,392.33	53.25	<0.001 *
Modifier concentration (B)	1,258,913.70	2	629,456.85	4.82	0.011 *
A × B	253,915.29	4	63,478.82	0.49	0.746
Error	7,830,128.98	60	130,502.15		

Note: The analysis included the nine modified groups; the control group (PMMA_Cnt) was not included. df, degrees of freedom; F, F statistic. * Statistically significant (*p* < 0.05).

**Table 10:** Mean Young’s modulus values according to modifier type and concentration (MPa; mean ± standard deviation).

Modifier Type	2%	3%	5%
SO	2559.29 ± 302.97 ^Aa^	2704.51 ± 364.19 ^Aa^	2206.07 ± 228.97 ^Aa^
PO	2105.22 ± 461.95 ^Aa^	2068.96 ± 604.56 ^Ba^	1924.26 ± 390.49 ^Aa^
LIP	1446.79 ± 153.07 ^Ba^	1447.88 ± 156.58 ^ca^	1178.49 ± 147.39 ^Ba^

Note: Within the same column, values not sharing a common uppercase letter differ significantly among modifier types; within the same row, values not sharing a common lowercase letter differ significantly among modifier concentrations (Bonferroni-adjusted pairwise comparisons, *p* < 0.05). SD, standard deviation.

**Table 11:** Descriptive statistics and Shapiro–Wilk normality test and between-group comparison results for Young’s modulus (MPa).

Group	Mean ± SD	Minimum	Maximum	Shapiro–Wilk Statistic	Shapiro–Wilk *p*	ANOVA F	*p*
PMMA_Cnt	2562.19 ± 197.68 ^ab^	2304.61	2830.29	0.953	0.759	15.783	<0.001
PMMA_SO_2	2559.29 ± 302.97 ^ab^	2164.30	2961.99	0.939	0.629		
PMMA_SO_3	2704.51 ± 364.19 ^a^	2261.85	3415.03	0.904	0.358		
PMMA_SO_5	2206.07 ± 228.97 ^abc^	1920.31	2585.62	0.961	0.819		
PMMA_PO_2	2105.22 ± 461.95 ^abc^	1226.01	2716.81	0.945	0.637		
PMMA_PO_3	2068.96 ± 604.56 ^bc^	1439.22	3417.59	0.846	0.068		
PMMA_PO_5	1924.26 ± 390.49 ^cd^	1314.62	2680.29	0.939	0.602		
PMMA_LIP_2	1446.79 ± 153.07 ^de^	1309.71	1760.20	0.819	0.063		
PMMA_LIP_3	1447.88 ± 156.58 ^de^	1158.40	1609.95	0.906	0.369		
PMMA_LIP_5	1178.49 ± 147.39 ^e^	969.14	1376.35	0.954	0.762		
Total	2025.06 ± 582.20	969.14	3417.59	–	–		

Note: Values are presented as mean ± standard deviation (SD). Groups that do not share a common superscript letter differ significantly according to Bonferroni-adjusted pairwise comparisons (*p* < 0.05); groups sharing at least one common letter are not significantly different. The overall between-group comparison was performed using one-way ANOVA because the homogeneity of variances assumption was satisfied. Normality was assessed using the Shapiro–Wilk test. SD, standard deviation.

**Table 12:** Two-way factorial analysis of variance results for Vickers hardness.

Source	Sum of Squares	df	Mean Square	F	*p*
Modifier type (A)	1360.83	2	680.42	93.40	<0.001 *
Modifier concentration (B)	20.08	2	10.04	1.38	0.259
A × B	51.44	4	12.86	1.77	0.146
Error	502.65	69	7.29		

Note: The analysis included the nine modified groups; the control group (PMMA_Cnt) was not included. df, degrees of freedom; F, F statistic. * Statistically significant (*p* < 0.05).

**Table 13:** Mean Vickers hardness values (HV) according to modifier type and modifier concentration (mean ± SD).

Modifier Type	2%	3%	5%
SO	18.03 ± 1.76 ^Aa^	18.61 ± 4.49 ^Aa^	19.73 ± 3.19 ^Aa^
PO	15.72 ± 2.11 ^Aa^	18.62 ± 2.99 ^Aa^	18.49 ± 2.52 ^Aa^
LIP	10.08 ± 2.97 ^Ba^	9.90 ± 1.26 ^Ba^	8.68 ± 1.13 ^Ba^

Note: Within the same column, values not sharing a common uppercase letter differ significantly among modifier types; within the same row, values not sharing a common lowercase letter differ significantly among modifier concentrations (Bonferroni-adjusted pairwise comparisons of estimated marginal means, *p* < 0.05). SD, standard deviation.

**Table 14:** Descriptive statistics, Shapiro–Wilk normality test, and between-group comparison results for Vickers hardness (HV).

Group	Mean ± SD	Minimum	Maximum	Shapiro–Wilk Statistic	Shapiro–Wilk *p*	Welch F	*p*
PMMA_Cnt	23.22 ± 3.41 ^a^	17.37	28.32	0.984	0.981	44.517	<0.001
PMMA_SO_2	18.03 ± 1.76 ^ab^	15.05	20.35	0.941	0.596		
PMMA_SO_3	18.61 ± 4.49 ^ab^	11.07	25.14	0.975	0.937		
PMMA_SO_5	19.73 ± 3.19 ^ab^	13.34	24.80	0.963	0.824		
PMMA_PO_2	15.72 ± 2.11 ^b^	13.04	18.10	0.861	0.098		
PMMA_PO_3	18.62 ± 2.99 ^ab^	13.38	22.93	0.934	0.522		
PMMA_PO_5	18.49 ± 2.52 ^ab^	14.49	20.67	0.889	0.354		
PMMA_LIP_2	10.08 ± 2.97 ^c^	5.22	16.49	0.942	0.571		
PMMA_LIP_3	9.90 ± 1.26 ^c^	8.17	12.24	0.942	0.604		
PMMA_LIP_5	8.68 ± 1.13 ^c^	7.24	11.17	0.873	0.131		
Total	15.93 ± 5.44	5.22	28.32	–	–		

Note: Values are presented as mean ± standard deviation (SD). Groups that do not share a common superscript letter differ significantly according to Tamhane’s T2 post hoc test (*p* < 0.05); groups sharing at least one common letter are not significantly different. Because Levene’s test indicated heterogeneity of variances, Welch’s ANOVA was used for the overall between-group comparison, followed by Tamhane’s T2 multiple-comparison test. Normality was assessed using the Shapiro–Wilk test. SD, standard deviation.

## Data Availability

The original contributions presented in this study are included in the article. Further inquiries can be directed to the corresponding author.

## Abbreviations

The following abbreviations are used in this manuscript:

PMMA	Poly(methyl methacrylate)
MMA	Methyl methacrylate
SO	4-styrenesulfonate (SO)
PO	5,5-Bis(diisopropoxyphosphoryl)pentyl methacrylate
LIP	5,5-Bis(diisopropoxyphosphoryl)pentyl lipoate
LA	α-Lipoic acid
Tiph	Tetraisopropyl methylenediphosphonate
Tiph-A	5,5-Bis(diisopropoxyphosphoryl)pentyl acetate
Tiph-OH	Tetraisopropyl (5-hydroxypentane-1,1-diyl)bis(phosphonate)
DCC	N,N′-Dicyclohexylcarbodiimide
DMAP	4-(Dimethylamino)pyridine
TGA	Thermogravimetric analysis
DTA	Differential thermal analysis
Td1, Td2	Early and main degradation temperatures
SEM	Scanning electron microscopy
